# Aerobic Capacity Is Related to Multiple Other Aspects of Physical Fitness: A Study in a Large Sample of Lithuanian Schoolchildren

**DOI:** 10.3389/fphys.2018.01797

**Published:** 2018-12-11

**Authors:** Tomas Venckunas, Brigita Mieziene, Arunas Emeljanovas

**Affiliations:** Institute of Sport Science and Innovations, Lithuanian Sports University, Kaunas, Lithuania

**Keywords:** children, adolescents, testing, musculoskeletal fitness, motor fitness, cardiorespiratory capacity

## Abstract

This study evaluated how aerobic capacity is related to performance in other aspects of health-related physical fitness among schoolchildren. The study involved >15,200 schoolchildren of both genders aged 11–18 years, who were tested with a reliable tests from Eurofit battery for most important aspects of exercise capacity and anthropometrics from 1992 to 2012. The analysis showed that aerobic capacity was weakly but significantly positively related to all other aspects of exercise abilities tested in all age groups for both genders. Variance of performance in agility shuttle run and standing broad jump were each explained by aerobic capacity the strongest (>10%), followed by weaker but still significant positive relation of aerobic capacity with the abilities in bent arm hang and abdominal curl tests (aerobic capacity explaining ∼6.5% of the variance of the performance in these tests), as well as in balance and flexibility tasks (aerobic capacity significantly explaining ∼3% of the variance). Thus, while aerobic capacity in schoolchildren of all ages and both genders can explain the performance in other aspects of physical fitness and especially leg muscle power, the percent of explained variance in the results of any these tests was not high and therefore aerobic capacity should be tested as a separate important fitness parameter which cannot be substituted by other tests from the Eurofit battery.

## Introduction

Health-related physical fitness is a multifactorial construct that encompasses cardio-respiratory (aerobic) capacity, muscular strength, speed/agility, balance and flexibility components (Huang and Malina, 2002). The level of physical fitness harbors important information about current and future cardio-vascular, skeletal and mental health (Catley and Tomkinson, 2013). In particular, aerobic capacity, which is the highest amount of oxygen consumed during maximal exercise in activities that uses the large muscle groups, is associated with a risk of developing metabolic syndrome or diabetes (Carnethon et al., 2003; Ruiz et al., 2009) as well as with a risk of cardiac events (Laukkanen et al., 2004). More than that, it has been shown that low aerobic capacity is not only a substantial risk factor for a vast range of modern diseases such as cancer, cardiovascular diseases (Antero-Jacquemin et al., 2018) and diabetes (Laine et al., 2017), but it is the most powerful predictor of overall mortality equally among healthy people and patients (Blair et al., 1989, 1995; Myers et al., 2002). It has been recognized that risk factors for modern chronic diseases have their origins in childhood and adolescence (Berenson et al., 1998; Katzmarzyk et al., 2001; Ebbeling et al., 2002; Ortega et al., 2008; Dwyer et al., 2009; Ruiz et al., 2009, 2016). It is metabolic health that is particularly strongly associated with aerobic capacity, and it has been reported that about 4% of schoolchildren in the United States already have metabolic syndrome (Welk et al., 2011). The sharp decrease in aerobic capacity among schoolchildren during the last decades (Tomkinson et al., 2003; Tomkinson and Olds, 2007; Venckunas et al., 2017) raise serious concerns about the health of the upcoming generations. Recent declines in children’s aerobic capacity have been attributed to increases in obesity (Albon et al., 2010) and to reduced physical activity (Huotari et al., 2010; Pahkala et al., 2013), which are interrelated (Lamboglia et al., 2013).

While aerobic capacity in schoolchildren has been found to be related to the abilities in explosive- and motor skill-demanding movements involving either the legs or arms (Okely et al., 2001), changes in various components of physical fitness during childhood and adolescence do not always change the same as aerobic capacity and in addition may depend on other factors such as gender (Catley and Tomkinson, 2013). In addition, it has been shown that various aspects of motor fitness in children are not following the same trend of change across decades (Runhaar et al., 2010). It is thus still unclear as to what extent cardio-respiratory fitness is related to muscle strength/power, balance and flexibility parameters among children and adolescents.

The aim of this study was to test the relationship of aerobic capacity with performance in other aspects of physical fitness among children and adolescents. We hypothesized that there would be a positive relationship of aerobic capacity with other aspects of fitness, indicative of the general dependence of fitness on activity levels. Declines in multiple aspects of fitness would then exacerbate the disease risk in lower fitness percentiles of schoolchildren. Moreover, if a significant relationship between cardio-respiratory (aerobic) capacity and other aspects of physical fitness is found, this would imply a substantial interplay between motor abilities and confirm the idea of the importance of comprehensive physical development as by sports practices to ensure general well-being through regular participation in active leisure time (Runhaar et al., 2010).

## Materials and Methods

### Participants

The study included data from participants from the three nationally representative cohort studies performed in Lithuania in the years 1992, 2002, and 2012 among 11- to 18-year-old schoolchildren. In total, 18,294 schoolchildren were recruited for testing. Only those who had their body weight and height measured, and completed a shuttle endurance test and at least one other test were included in the analysis. The final number included 15,213 participants (7,608 boys and 7,605 girls). The distribution of numbers of participants for both genders across age groups and the three decades are presented in Table 1.

**Table 1:** The distribution of number of participants (*n*) across age groups and decades.

	Boys					Girls				
	
	11–12 years	13–14 years	15–16 years	17–18 years	Total	11–12 years	13–14 years	15–16 years	17–18 years	Total
**1992**										
*n*	880	922	551	232	2585	870	916	649	369	2804
*% within decade*	*34.0*	*35.7*	*21.3*	*9.0*	*100.0*	*31.0*	*32.7*	*23.1*	*13.2*	*100.0*
**2002**										
*n*	805	696	635	418	2554	758	727	620	438	2543
*% within decade*	*31.5*	*27.3*	*24.9*	*16.4*	*100.0*	*29.8*	*28.6*	*24.4*	*17.2*	*100.0*
**2012**										
*n*	411	691	761	606	2469	412	600	716	530	2258
*% within decade*	*16.6*	*28.0*	*30.8*	*24.5*	*100.0*	*18.2*	*26.6*	*31.7*	*23.5*	*100.0*

### Procedures

The study was carried out in accordance with the recommendations listed in Declaration of Helsinki. The protocol was approved by the Lithuanian Bioethics Committee (permission no. BE-2-45). Informed consent was obtained from all participants, and written informed consent was also provided by their parents/guardians. Data were collected during the spring in all three time points. Physical fitness evaluations using the Eurofit test battery and anthropometrical measurements were taken by a team of qualified testers—graduates of our sports university—who had all been trained by the same chief investigator across the three decades. Schools were selected from a national registry including major cities and districts across the country (*n* = 14, *n* = 14 and *n* = 19 in 1992, 2002, and 2012, respectively). Schools were classified into groups by type of location (urban or rural). Selection was made by randomly selecting school code from the boxes of urban and rural school codes, with each school having an equal probability of selection. Schoolchildren were selected based on grade (from 5th to 12th) and were included if healthy and attended physical education classes at the time of the study. Physical fitness tests were performed wearing gym attire. Body weight and height were measured before the tests. To avoid fatigue, tests were split into two non-consecutive days within 1 week. Tests were administered and performed by the children in the following order: day 1—Flamingo balance, sit-and-reach, standing broad jump, sit-ups, bent arm hang and 10 × 5 m agility shuttle run; day 2—endurance 20 m shuttle run (Venckunas et al., 2017).

Testing procedures were standardized among testers before testing in each decade. Reliability of Eurofit tests has been investigated in a population of young subjects (Tsigilis et al., 2002) in whom intraclass correlation revealed satisfactory coefficients >0.70 for the tests used in the current study (*r* was 0.57 for the plate-tapping test, the one which had not been included into the current study). All testing procedures were meticulously explained to children on the day of testing. Testing equipment calibration was performed periodically (before each testing session in each of the schools) in the university settings. Measurements were taken in accordance with standard methodology for anthropometrics and Eurofit physical fitness tests.

### Physical Fitness Tests

The different components of physical fitness—balance, flexibility, muscular strength, power and endurance, agility and cardio-respiratory fitness—were assessed by the standardized Eurofit test battery as described previously (Venckunas et al., 2017).

In brief, ***Flamingo balance*** test measures static balancing ability by the number of attempts required to complete the total of balancing standing on the rod for 1 min on one foot. ***Sit-and-reach*** test measures lower body flexibility while attempting to reach forward as far as possible keeping knees straight in a sitting position. ***Standing broad jump*** measures jumping distance from a standing start (‘frog leap‘). ***Sit-up test*** measures abdominal muscles function as number of sit-ups completed from lying position (knees bent at a 90°) in 30 s. ***Bent arm hang*** ***test*** measures function of upper muscles as time which participants are able to sustain the both hand grasp hang with the chin above the bar. ***Agility shuttle run*** measures the time required to complete 50 m shuttle run test from a standing start during which the participants run forth and back five times to complete five 10 m laps. ***Endurance shuttle run*** measures cardiorespiratory fitness (aerobic capacity) as the number of stages completed during every-minute increasing pace of 20 m shuttle run test which begins with walking and proceeds to running. The result of the test provides a valid estimate of treadmill maximal oxygen uptake in young adults (Paradisis et al., 2014).

A standard warm-up (mostly running and dynamic stretching exercises) for 7–8 min was carried out before testing. Before each of the tests, the participants were given a try, and the importance of concentration and maximal efforts was reminded. The better result of two attempts was recorded in sit-and-reach and standing broad jump tests, while in other tests only one successful attempt was allowed. All tests have been conducted indoors (school’s gym) in the comfortable sporting attire; jumping and running tests were carried out on wooden non-slippery floor.

### Anthropometric Measurements

Barefoot stature was measured to the nearest 0.1 cm, and body weight was measured to the nearest 0.1 kg when participants were wearing minimal clothing. The body mass index (BMI) was calculated as body weight per height squared (kg/m^2^).

### Statistical Analysis

IBM SPSS Statistics v. 24.0 for Windows (IBM Corp., Armonk, NY, United States) was used for data processing. Normality tests for each gender and age group were applied to identify outliers, which were subsequently excluded. Means, standard deviations and frequencies were calculated using descriptive statistics. Participants were allocated into quintiles according to the result of their endurance shuttle run test. Classification of schoolchildren to cardio-vascular fitness quintiles of “very low” to “very high” fitness was based on previous studies (Blair et al., 1989, 1995; Myers et al., 2002; Catley and Tomkinson, 2013). For the analysis, data from the three different decades were pooled. A generalized linear model univariate analysis using BMI and decade (the year in which the measurements were taken) as covariates was performed to test for the differences in the performance in other physical fitness tests between the quintiles of cardio-respiratory fitness level. Analyses for boys and girls were performed separately. A Bonferroni *post hoc* test was used for multiple comparisons, and two-sided *p*-values of <0.05 were considered statistically significant.

## Results

Quintiles of the number of completed endurance shuttle run stages revealed that aerobic capacity increased with age in boys but remained similar across age groups in girls (Table 2). Fewer subjects were found at the two highest quintiles in all age groups and in both genders. Aerobic capacity was positively related to all other aspects of physical fitness tested in all age groups and both genders of schoolchildren. Aerobic capacity was most strongly related to performance in agility shuttle run and standing broad jump, where it explained >10% of the variance in performance in these tests; then followed bent arm hang time and number of abdominal curls, the results of each of which were explained by ∼6–7% of the variance in aerobic capacity; the performance in Flamingo balance and sit-and-reach flexibility tests were each explained by ∼3% of the variance in aerobic capacity.

**Table 2:** Quintiles of number of completed endurance shuttle run stages (proxy for aerobic capacity) in schoolchildren.

Gender	Age, years	Quintiles of aerobic capacity				
		
		1st <20% (very low)	2nd 20–40% (low)	3rd 40–60% (average)	4th 60–80% (high)	5th >80% (very high)
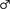	11–12 (*n* = 2204)	≤4 (*n* = 626; 28.4%)	5 (*n* = 531; 24.1%)	6 (*n* = 397; 18%)	7–8 (*n* = 320; 14.5%)	≥9 (*n* = 330; 15%)
	13–14 (*n* = 2412)	≤4 (*n* = 644; 26.7%)	5–6 (*n* = 498; 20.6%)	7 (*n* = 550; 22.8%)	8–9 (*n* = 328; 13.6%)	≥10 (*n* = 392; 16.3%)
	15–16 (*n* = 1995)	≤5 (*n* = 477; 23.9%)	6–7 (*n* = 421; 21.1%)	8 (*n* = 440; 22.1%)	9 (*n* = 400; 20.1%)	≥10 (*n* = 257; 12.9%)
	17–18 (*n* = 1315)	≤5 (*n* = 314; 23.9%)	6–7 (*n* = 288; 21.9%)	8 (*n* = 299; 22.7%)	9 (*n* = 275; 20.9%)	≥10 (*n* = 139; 10.6%)
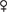	11–12 (*n* = 2130)	≤3 (*n* = 661; 31%)	4 (*n* = 440; 20.7%)	5 (*n* = 395; 18.5%)	6 (*n* = 281; 13.2%)	≥7 (*n* = 353; 16.6%)
	13–14 (*n* = 2328)	≤3 (*n* = 619; 26.6%)	4 (*n* = 461; 19.8%)	5 (*n* = 479; 20.6%)	6 (*n* = 370; 15.9%)	≥7 (*n* = 399; 17.1%)
	15–16 (*n* = 2027)	≤3 (*n* = 550; 27.1%)	4 (*n* = 483; 23.8%)	5 (*n* = 436; 21.5%)	6 (*n* = 286; 11.8%)	≥7 (*n* = 272; 13.4%)
	17–18 (*n* = 1367)	≤3 (*n* = 428; 31.3%)	4 (*n* = 315; 23%)	5 (*n* = 279; 20.4%)	6 (*n* = 186; 13.6%)	≥7 (*n* = 159; 11.6%)


Agility was significantly related to aerobic capacity level in both genders across all age groups. BMI had a marginal effect for this relationship, while the decade of study was a significant covariate in all age groups for both genders (Table 3). Bent arm hang time differed between quintiles of aerobic capacity in both genders and across all age groups. BMI was a significant co-factor in this measure for all age groups and both genders (Table 4). Numbers of abdominal curls completed in 30 s differed between quintiles of aerobic capacity in both genders and across all age groups. BMI had a small but significant effect on this relationship (except for the oldest group of boys), and decade was a significant covariate for this relationship in all age groups in both genders (Table 5). Standing broad jump results differed between quintiles of aerobic capacity in both genders and across all age groups, with a minor effect of BMI and decade (Table 6). Balancing ability was moderately but significantly affected by aerobic capacity level in both genders and across all age groups, with a minor effect of BMI and some more substantial effect of decade on the relationship (Table 7). Lower body flexibility was moderately but significantly interrelated to aerobic capacity level in both genders and across all age groups except for the oldest (17- to 18-year-old) boys group in which were no significant differences between aerobic capacity quintiles in sit-and-reach flexibility (Table 8); small but significant effects of BMI and decade were detected for this relationship (Table 8).

**Table 3:** Agility shuttle run(s) in schoolchildren of different aerobic capacity levels.

Gender	Age, years	Quintiles of aerobic capacity					Quintile’s effect ($\eta_{p}^{2}$)	Covariates’ effect ($\eta_{p}^{2}$)	
		1st <20% (very low)	2nd 20–40% (low)	3rd 40–60% (average)	4th 60–80% (high)	5th >80% (very high)		Body mass index	Decade
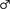	11–12 (*n* = 1989)	22.1 (2.1) ^b-e^	21.5 (1.8) ^d,e^	21.5 (1.7) ^d,e^	21.2 (1.6) ^e^	20.8 (1.4)	0.136 ^∗∗∗^	0.004 ^∗∗^	0.121 ^∗∗∗^
	13–14 (*n* = 2217)	21.2 (2.3)^b-e^	20.8 (1.8) ^c-e^	20.5 (1.8) ^d,e^	20.4 (1.5) ^e^	20.0 (1.2)	0.097 ^∗∗∗^	0.004 ^∗∗^	0.070 ^∗∗∗^
	15–16 (*n* = 1872)	20.3 (2.1) ^b-e^	19.9 (1.8) ^c-e^	19.8 (1.7) ^e^	19.3 (1.9)	19.4 (1.5)	0.105 ^∗∗∗^	<0.001	0.102 ^∗∗∗^
	17–18 (*n* = 1199)	20.3 (2.3)^b-e^	19.4 (1.6) ^d,e^	19.2 (1.6) ^e^	18.7 (1.9)	18.8 (1.6)	0.109 ^∗∗∗^	<0.001	0.025 ^∗∗∗^
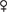	11–12 (*n* = 885)	23.3 (2.2) ^b-e^	23.1 (1.8) ^c-e^	22.5 (1.7) ^d,e^	22.3 (1.5) ^e^	22.0 (1.4)	0.139 ^∗∗∗^	0.001	0.083 ^∗∗∗^
	13–14 (*n* = 2140)	22.5 (2.1)^b-e^	22.2 (1.8) ^c-e^	21.8 (1.7) ^d,e^	21.7 (1.6) ^e^	21.2 (1.5)	0.101 ^∗∗∗^	0.005 ^∗∗^	0.065 ^∗∗∗^
	15–16 (*n* = 1889)	22.6 (2.0)^b-e^	21.8 (1.9) ^d,e^	21.6 (1.6) ^d,e^	21.3 (1.5) ^e^	20.9 (1.3)	0.112 ^∗∗∗^	0.004 ^∗∗^	0.020 ^∗∗∗^
	17–18 (*n* = 1290)	22.4 (1.9)^b-e^	21.7 (1.8) ^d,e^	21.4 (1.6) ^d,e^	20.9 (1.7)	20.7 (1.4)	0.114 ^∗∗∗^	0.018 ^∗∗^	0.016 ^∗∗∗^

*Data presented as mean (SD). Faster times mean better agility. ^*b,c,d,e*^ differ at *p* < 0.05 from 2nd, 3rd, 4th, and 5th quintiles, respectively. ^∗∗^*p* < 0.01, ^∗∗∗^*p* < 0.001, respectively*.

**Table 4:** Bent arm hang time(s) in schoolchildren of different aerobic capacity levels.

Gender	Age, years	Quintiles of aerobic capacity					Quintile’s effect ($\eta_{p}^{2}$)	Covariates’ effect ($\eta_{p}^{2}$)	
		1st <20% (very low)	2nd 20–40% (low)	3rd 40–60% (average)	4th 60–80% (high)	5th >80% (very high)		Body mass index	Decade
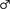	11–12 (*n* = 1983)	13.0 (10.7) ^b-e^	17.8 (12.0) ^d,e^	20.9 (13.3) ^d,e^	25.0 (14.3) ^e^	28.8 (15.7)	0.112 ^∗∗∗^	0.037 ^∗∗∗^	0.006 ^∗∗^
	13–14 (*n* = 2198)	18.0 (14.7) ^b-e^	20.2 (12.8) ^c-e^	26.4 (14.1) ^d,e^	28.4 (13.5) ^e^	31.9 (15.5)	0.073 ^∗∗∗^	0.018 ^∗∗∗^	<0.001
	15–16 (*n* = 1855)	24.6 (14.3) ^b-e^	30.6 (15.0) ^d,e^	33.6 (15.8) ^e^	37.8 (15.7)	39.0 (16.3)	0.056 ^∗∗∗^	0.017 ^∗∗∗^	0.008 ^∗∗∗^
	17–18 (*n* = 1198)	25.0 (15.5) ^b-e^	32.7 (18.2) ^d,e^	34.3 (18.8)	38.7 (17.3)	42.9 (19.8)	0.045 ^∗∗∗^	0.037 ^∗∗∗^	0.022 ^∗∗∗^
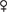	11–12 (*n* = 838)	7.5 (6.3) ^b-e^	10.5 (8.4) ^c-e^	11.7 (8.1) ^e^	12.1 (8.1) ^e^	16.6 (9.5)	0.106 ^∗∗∗^	0.031 ^∗∗∗^	0.009 ^∗∗^
	13–14 (*n* = 2029)	8.4 (7.2) ^b-e^	11.1 (8.9) ^e^	12.8 (9.2) ^e^	12.4 (9.2) ^e^	16.2 (9.6)	0.054 ^∗∗∗^	0.037 ^∗∗∗^	0.001
	15–16 (*n* = 1814)	10.0 (8.4) ^b-e^	11.9 (9.2) ^d,e^	12.7 (9.1) ^e^	14.7 (9.9)	16.2 (9.7)	0.039 ^∗∗∗^	0.057 ^∗∗∗^	0.002
	17–18 (*n* = 1177)	9.3 (8.4) ^c-e^	11.2 (8.1) ^d,e^	12.5 (9.2) ^e^	15.5 (11.0) ^e^	16.3 (12.2)	0.061 ^∗∗∗^	0.082 ^∗∗∗^	0.009 ^∗∗^

*Data presented as mean (SD). ^*b,c,d,e*^ differ at *p* < 0.05 from 2nd, 3rd, 4th, and 5th quintiles, respectively. ^∗^*p* < 0.05, ^∗∗^*p* < 0.01, ^∗∗∗^*p* < 0.001, respectively*.

**Table 5:** Abdominal curls (no. per 30 s) in schoolchildren of different aerobic capacity levels.

Gender	Age, years	Quintiles of aerobic capacity					Quintile’s effect ($\eta_{p}^{2}$)	Covariates’ effect ($\eta_{p}^{2}$)	
		1st <20% (very low)	2nd 20–40% (low)	3rd 40–60% (average)	4th 60–80% (high)	5th >80% (very high)		Body mass index	Decade
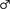	11–12 (*n* = 2020)	22.8 (4.3) ^b-e^	24.1 (4.1) ^d,e^	25.0 (3.8) ^d,e^	25.0 (3.8) ^e^	26.0 (3.4)	0.076 ^∗∗∗^	0.007 ^∗∗∗^	0.020 ^∗∗∗^
	13–14 (*n* = 2228)	24.6 (4.8) ^b-e^	25.7 (4.6) ^d,e^	26.6 (4.1) ^d,e^	27.1 (3.8) ^e^	28.0 (3.8)	0.059 ^∗∗∗^	0.007 ^∗∗∗^	0.007 ^∗∗∗^
	15–16 (*n* = 1870)	26.5 (4.5) ^b-e^	27.3 (4.0) ^d,e^	27.9 (4.1) ^e^	28.8 (3.8) ^e^	29.2 (3.5)	0.062 ^∗∗∗^	0.003 ^∗^	0.014 ^∗∗∗^
	17–18 (*n* = 1219)	27.3 (5.1) ^b-e^	28.6 (4.3) ^d,e^	28.7 (3.9) ^e^	29.5 (3.9)	30.2 (4.3)	0.060 ^∗∗∗^	0.001	0.024 ^∗∗∗^
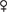	11–12 (*n* = 899)	21.1 (4.1) ^b-e^	21.8 (3.8) ^d,e^	22.0 (3.6) ^e^	22.6 (4.0)	23.3 (3.5)	0.055 ^∗∗∗^	0.010 ^∗∗^	0.026 ^∗∗∗^
	13–14 (*n* = 2138)	21.9 (4.7) ^b-e^	23.2 (4.3) ^e^	23.8 (4.0) ^e^	23.5 (3.9) ^e^	24.6 (3.7)	0.056 ^∗∗∗^	0.012 ^∗∗∗^	0.025 ^∗∗∗^
	15–16 (*n* = 1906)	23.4 (4.3) ^b-e^	24.1 (4.2) ^d,e^	24.1 (3.8) ^d,e^	24.7 (3.8)	24.8 (3.7)	0.027 ^∗∗∗^	0.007 ^∗∗∗^	0.022 ^∗∗∗^
	17–18 (*n* = 1287)	24.3 (4.3) ^b-e^	24.9 (4.0) ^d,e^	25.4 (3.6) ^e^	26.1 (3.9)	26.4 (3.7)	0.069 ^∗∗∗^	0.011 ^∗∗∗^	0.058 ^∗∗∗^

*Data presented as mean (SD). ^*b,c,d,e*^ differ at *p* < 0.05 from 2nd, 3rd, 4th, and 5th quintiles, respectively. ^∗^*p* < 0.05, ^∗∗^*p* < 0.01, ^∗∗∗^*p* < 0.001, respectively*.

**Table 6:** Standing broad jump (cm) in schoolchildren of different aerobic capacity levels.

Gender	Age, years	Quintiles of aerobic capacity					Quintile’s effect ($\eta_{p}^{2}$)	Covariates’ effect ($\eta_{p}^{2}$)	
		1st <20% (very low)	2nd 20–40% (low)	3rd 40–60% (average)	4th 60–80% (high)	5th >80% (very high)		Body mass index	Decade
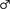	11–12 (*n* = 2033)	155.3 (20.7) ^b-e^	169.1 (17.7) ^d,e^	170.1 (17.8) ^d,e^	174.4 (17.0) ^e^	178.8 (16.1)	0.138 ^∗∗∗^	0.004 ^∗∗^	0.007 ^∗∗∗^
	13–14 (*n* = 2236)	181.3 (26.1) ^b-e^	185.1 (23.5) ^c-e^	195.5 (21.7) ^d,e^	195.6 (21.2) ^e^	201.9 (18.6)	0.100 ^∗∗∗^	0.004 ^∗∗^	0.008 ^∗∗∗^
	15–16 (*n* = 1875)	201.7 (27.2) ^b-e^	211.3 (23.5) ^c-e^	215.7 (22.3) ^e^	222.9 (20.1) ^e^	226.1 (17.5)	0.120 ^∗∗∗^	0.002 ^∗^	0.007 ^∗∗∗^
	17–18 (*n* = 1217)	210.9 (33.8) ^b-e^	227.8 (24.3) ^e^	229.5 (22.0)	234.1 (20.6)	238.0 (19.0)	0.105 ^∗∗∗^	0.002	0.001
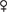	11–12 (*n* = 900)	141.2 (18.5) ^b-e^	148.5 (17.4) ^c-e^	153.6 (15.3) ^d,e^	159.5 (15.6) ^e^	163.9 (15.6)	0.123 ^∗∗∗^	0.010 ^∗∗^	<0.001
	13–14 (*n* = 2157)	151.7 (20.1) ^b-e^	161.3 (18.4) ^c-e^	166.8 (17.9) ^e^	170.3 (15.6) ^e^	176.0 (17.0)	0.125 ^∗∗∗^	0.015 ^∗∗∗^	<0.001
	15–16 (*n* = 1914)	158.7 (21.2) ^b-e^	166.1 (20.4) ^c-e^	170.5 (19.5) ^d,e^	176.9 (18.4) ^e^	182.1 (14.6)	0.102 ^∗∗∗^	0.013 ^∗∗∗^	<0.001
	17–18 (*n* = 1265)	162.3 (20.8) ^b-e^	169.2 (17.4) ^d,e^	173.4 (16.7) ^d,e^	182.4 (17.3) ^e^	188.5 (19.8)	0.130 ^∗∗∗^	0.053 ^∗∗∗^	<0.001

*Data presented as mean (SD). ^*b,c,d,e*^ differ at *p* < 0.05 from 2nd, 3rd, 4th, and 5th quintiles, respectively. ^∗^*p* < 0.05, ^∗∗^*p* < 0.01, ^∗∗∗^*p* < 0.001, respectively*.

**Table 7:** Flamingo balance (no. of attempts in 1 min) in schoolchildren of different aerobic capacity levels.

Gender	Age, years	Quintiles of aerobic capacity					Quintile’s effect ($\eta_{p}^{2}$)	Covariates’ effect ($\eta_{p}^{2}$)	
		1st <20% (very low)	2nd 20–40% (low)	3rd 40–60% (average)	4th 60–80% (high)	5th >80% (very high)		Body mass index	Decade
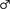	11–12 (*n* = 2052)	13.1 (6.0) ^b-e^	12.5 (5.5) ^d,e^	12.3 (5.4) ^d,e^	12.0 (5.1)	11.4 (4.9)	0.030 ^∗∗∗^	0.013 ^∗∗∗^	0.047 ^∗∗∗^
	13–14 (*n* = 2255)	10.7 (6.0) ^d,e^	11.4 (6.0) ^d,e^	11.3 (5.5) ^d,e^	10.8 (5.1)	10.9 (5.0)	0.025 ^∗∗∗^	0.011 ^∗∗∗^	0.072 ^∗∗∗^
	15–16 (*n* = 1905)	10.3 (5.2) ^c-e^	9.9 (5.2) ^e^	10.3 (5.2) ^e^	10.0 (5.0)	10.0 (4.6)	0.022 ^∗∗∗^	0.003 ^∗∗^	0.067 ^∗∗∗^
	17–18 (*n* = 1238)	10.3 (4.9) ^d,e^	9.9 (5.0) ^d,e^	9.8 (4.7) ^e^	8.7 (4.5)	8.5 (4.8)	0.032 ^∗∗∗^	0.002	0.018 ^∗∗∗^
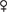	11–12 (*n* = 892)	12.9 (6.3) ^c-e^	12.7 (5.5) ^d,e^	12.8 (5.6)	12.3 (5.6)	13.6 (5.5)	0.022 ^∗∗∗^	0.022 ^∗∗∗^	0.089 ^∗∗∗^
	13–14 (*n* = 2190)	11.8 (5.9) ^b-e^	11.1 (5.4) ^d,e^	11.4 (5.5) ^e^	11.6 (5.3)	11.0 (4.9)	0.044 ^∗∗∗^	0.021 ^∗∗∗^	0.143 ^∗∗∗^
	15–16 (*n* = 1946)	10.3 (5.2) ^c-e^	10.1 (5.4) ^c-e^	9.7 (5.1)	10.2 (5.1)	10.8 (5.0)	0.029 ^∗∗∗^	0.003 ^∗^	0.130 ^∗∗∗^
	17–18 (*n* = 1301)	10.2 (4.7) ^b-e^	9.4 (4.8) ^d,e^	9.5 (5.2)	9.1 (4.6)	9.6 (4.8)	0.039 ^∗∗∗^	0.020 ^∗∗∗^	0.099 ^∗∗∗^

*Data presented as mean (SD). The lower number of attempts means better balance. ^*b,c,d,e*^ differ at *p* < 0.05 from 2nd, 3rd, 4th, and 5th quintiles, respectively. ^∗^*p* < 0.05, ^∗∗^*p* < 0.01, ^∗∗∗^*p* < 0.001, respectively*.

**Table 8:** Sit-and-reach flexibility (cm) in schoolchildren of different aerobic capacity levels.

Gender	Age, years	Quintiles of aerobic capacity					Quintile’s effect ($\eta_{p}^{2}$)	Covariates’ effect ($\eta_{p}^{2}$)	
		1st <20% (very low)	2nd 20–40% (low)	3rd 40–60% (average)	4th 60–80% (high)	5th >80% (very high)		Body mass index	Decade
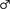	11–12 (*n* = 1993)	15.4 (5.6) ^b-e^	17.4 (5.6) ^d^	18.1 (5.4) ^d^	19.7 (5.7)	19.3 (5.2)	0.034 ^∗∗∗^	0.007 ^∗∗∗^	0.017 ^∗∗∗^
	13–14 (*n* = 2198)	17.2 (7.0) ^b-e^	18.6 (7.0) ^d,e^	20.9 (6.8)	21.1 (6.7)	21.8 (6.9)	0.026 ^∗∗∗^	0.024 ^∗∗∗^	0.013 ^∗∗∗^
	15–16 (*n* = 1843)	18.8 (8.3) ^b-e^	21.9 (7.7) ^e^	23.8 (7.6)	24.7 (8.1)	25.9 (7.8)	0.033 ^∗∗∗^	0.021 ^∗∗∗^	0.042 ^∗∗∗^
	17–18 (*n* = 1202)	22.3 (9.4)	24.1 (8.1)	24.5 (8.4)	25.6 (8.3)	27.3 (8.2)	0.008	0.015 ^∗∗∗^	0.057 ^∗∗∗^
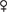	11–12 (*n* = 883)	17.9 (5.8) ^b-e^	19.2 (5.7) ^d,e^	20.4 (6.2)	21.2 (4.9)	20.9 (6.3)	0.036 ^∗∗∗^	0.021 ^∗∗∗^	0.001
	13–14 (*n* = 2113)	21.5 (6.7) ^b-e^	23.7 (6.5) ^d,e^	25.0 (6.3)	25.7 (6.0)	25.4 (5.9)	0.041 ^∗∗∗^	0.010 ^∗∗∗^	0.005 ^∗∗^
	15–16 (*n* = 1926)	23.5 (8.0) ^b-e^	25.8 (6.9)	26.9 (6.3)	28.0 (6.6)	28.8 (6.0)	0.029 ^∗∗∗^	0.019 ^∗∗∗^	0.043 ^∗∗∗^
	17–18 (*n* = 1298)	23.4 (8.7) ^b-e^	26.1 (7.3)	28.3 (6.6)	28.7 (6.0)	29.8 (6.2)	0.038 ^∗∗∗^	0.018 ^∗∗∗^	0.056 ^∗∗∗^

*Data presented as mean (SD). ^*b,c,d,e*^ differ at *p* < 0.05 from 2nd, 3rd, 4th, and 5th quintiles, respectively. ^∗^*p* < 0.05, ^∗∗^*p* < 0.01, ^∗∗∗^*p* < 0.001, respectively*.

## Discussion

Our analysis of the data collected over three decades revealed that aerobic capacity was weakly positively related with all aspects of exercise abilities tested in all age groups for both genders of children. The agility shuttle run and standing broad jump results were most strongly related to aerobic capacity. Part of this relatively strong relationship could be because of the similarity of movement pattern between shuttle endurance and agility shuttle run, and involvement of the same muscle groups (leg extensors) that are critical for locomotion in these tests. The endurance shuttle run, agility shuttle run and jumping are all weight-bearing exercises, as well as execution of all three requires acceleration (propulsion) and deceleration (landing) whole body. Therefore, both intrinsic muscular characteristics and proficiency in movement patterns such as synchronization of leg and arm swings in single leg (running) or both leg jumps to gain efficient momentum might have affected the results in the performance in these three tests (though to probably a different extent) compared with the influence on the performance in other tests. It is indeed that measures of anaerobic power such as a jumping ability explain considerable variation in endurance performance which clearly suggests some common shared physiologic mechanism (Houmard et al., 1991; Sinnett et al., 2001). Somewhat weaker but still significantly positively related to aerobic capacity were the results of bent arm hang, abdominal curls, balance, and flexibility tasks. Bent arm hang time and number of abdominal curls, representing upper body muscle fitness, were each explained by ∼6–7% of the variation in aerobic capacity. Differences in aerobic capacity explained only ∼3% of the variance in balance and lower body flexibility, even though lower body musculature is largely responsible for performance in both of these tests and the shuttle run test.

The positive interrelation between aerobic capacity and other physical capacities suggests that each of the applied tests from the Eurofit (or probably any other) battery has its own value in estimating ‘overall fitness’ of the individual. Even though at the highest level of multidisciplinary athletes there could be some trade-offs in performance between power and endurance events (Van Damme et al., 2002), this does not look to be true for the general non-athletic young population. On another end of age spectrum, it seems that not only endurance, but also speed-power training could somewhat ‘compensate’ for the lack of aerobic training stimulus and thus improve cardiovascular capacity as evidenced in the study of master athletes from different track-and-field disciplines (Kusy and Zieliński, 2014). It could be argued of course that it is not the parallel change in different aspects of physical fitness (i.e., comprehensive development of all aspect of exercise capacity) that is of primary importance and should be pursued but rather at least minimal level of any of the exercise capacities that is essential for a good quality of life and long years of independent subsistence. This seems to be especially relevant to the cardiorespiratory fitness as former elite endurance athletes have not increased life expectancy (van Saase et al., 1990; Farahmand et al., 2003; Sanchis-Gomar et al., 2011; Kettunen et al., 2015; Antero-Jacquemin et al., 2018) but also better life quality even long after discontinuation of athletic career (Bäckmand et al., 2010). However, as it is not only the endurance type of training bring about the beneficial adaptations (Bäckmand et al., 2010; Antero-Jacquemin et al., 2018), it could be recommended that in fact any of the vigorous exercise or any sports activity should be promoted among children.

The significant effect of the decade in which the testing was conducted indirectly supports the importance of recently changed activity patterns among schoolchildren as world trends show physical activity is on constant decline (Sallis et al., 2016). Lithuanian schoolchildren have also shown a continual decrease in physical activity levels during the period from 1998 to 2010 (Currie et al., 2012), which could well be the major reason of the declining aerobic capacity over decades in our cohort (Venckunas et al., 2017). Indeed, there is strong evidence to suggest that exercise capacity is largely determined by the level of physical activity (Huang and Malina, 2002; Huotari et al., 2010; Pahkala et al., 2013), even at a very early age (Lintu et al., 2016; Leppänen et al., 2017). For instance, it was reported that in 6- to 8-year-old children, physical activity and static balance were both linked to aerobic capacity (Lintu et al., 2016). Importantly, health-related behaviors including physical activity are to a significant extent transferred between school age and adulthood (Kelder et al., 1994; Telama et al., 1996). It is of interest that aerobic capacity in both genders of schoolchildren has also been found to be related to efficacy in explosive- and motor skill-demanding movements involving either the upper or lower musculature (Okely et al., 2001). Therefore, the idea that prevention of the decline in motor fitness in children is important for the overall engagement of kids into different exercise activities (Runhaar et al., 2010) is so relevant, and training various aspects of health related physical fitness via, e.g., participation in sports activities might prove indispensable for the accumulation of health capital.

The importance of aerobic capacity is well recognized at a young age already. During the school ages, low aerobic capacity is associated with an increased risk of cardio-vascular disease (Ruiz et al., 2009). Furthermore, after adjustment for age, ethnicity, gender, smoking, and family history of diabetes, hypertension, or premature myocardial infarction, it was shown that young adults with low cardio-vascular fitness (1st quintile) were threefold to sixfold more likely to develop diabetes, hypertension and metabolic syndrome than participants with high fitness (4th and 5th quintiles) during a 15-year period (Carnethon et al., 2003). Perhaps even more importantly, cardio-vascular fitness is the most powerful predictor of overall mortality among healthy people and cardiopulmonary patients (Blair et al., 1989, 1995; Myers et al., 2002).

The increase in aerobic capacity with age in our cohort was evident in boys but not girls, which is consistent with findings of other authors where non-athletic girls improved their aerobic capacity during maturation much slower than did boys (Catley and Tomkinson, 2013) while aerobic capacity in preadolescent schoolboys improves very slowly (De Miguel-Etayo et al., 2014). Our results for the distribution of endurance shuttle run lap numbers between quintiles are largely similar to data for Australian schoolchildren collected during a similar period (Okely et al., 2001; Catley and Tomkinson, 2013) and also those pooled from 50 countries (Tomkinson et al., 2017), implying that the data are representative of larger populations than those of particular regions.

### Limitations

We applied the progressive shuttle run (“beep”) test for measuring aerobic capacity, which in the later stages requires substantial muscle power for repeated acceleration and deceleration of the body. It could be that flat running in a circle (i.e., a stadium oval), such as the Cooper test, would produce different results, as partially reflected in the study on Australian children and adolescents where the beep test and flat 1-mile running results showed some differences across ages in that flat running seemingly improved less than the shuttle run for both genders (Catley and Tomkinson, 2013). However, as is usually the case with not well-trained subjects, the problems of proper pacing would have precluded a self-paced running field test from being really informative.

The relationships detected in the current study may to some extent also reflect the level and pattern of engagement in of part of the participants in physical activities and sports where increased participation would be associated with superior performance in the tests because of the enhanced physiological functions as a consequence of adaptation to regular training and higher competitive/motivational levels via learning effects during activities. This would be expected as those children who are physically active do not usually undertake one particular sport or concentrate on single event in that sport until the age of roughly 16 years but are rather encouraged by the physical education teachers and/or coaches to be involved in different exercise activities and learn many sports, which is (allegedly) a good relict of the Soviet time sports education culture in Lithuania. Consequently, it could be speculated that such comprehensive development ought to improve their overall fitness and most aspects of exercise capacities, while those schoolchildren leading largely sedentary life would not benefit from sports in any of the conditioning aspects. This scenario would create ‘scissors’ in the population tested, but as long as we alas cannot investigate this hypothesis in the frame of the current cohort because the information on the physical activity levels or sports participation is completely lacking in the database, this remains to be proved in other studies.

Our results do not necessary suggest a direct causal relationship of aerobic capacity with other exercise abilities. It might well be that increased activity levels augment aerobic capacity along with other physical abilities. However, as aerobic capacity was shown to be consistently associated with a vast range of other exercise abilities in both genders and all age groups of schoolchildren, this highlights its importance in overall motor abilities and overall well-being. Testing for aerobic capacity in schoolchildren and upgrading its level to higher quintiles by regular training could allow for improvements in other physical abilities, in addition to enabling continued interest and involvement in daily exercise activities including sports. Therefore, physical education teachers are encouraged to refer to the presented norms of aerobic capacity to monitor children and especially guide those in the lower quintiles (Myers et al., 2002) toward lifestyle modifications through implementation of individualized training and physical education programs.

## Conclusion

Aerobic capacity in all age groups and in both genders of schoolchildren is positively related to all other aspects of physical fitness, with the relationship being strongest with lower body muscular power.

## Author Contributions

TV, BM, and AE designed the study, and collected and analyzed the data. All authors contributed to interpretation of the data, drafting, and revising the manuscript, and approved the final version of the manuscript.

## Conflict of Interest Statement

The authors declare that the research was conducted in the absence of any commercial or financial relationships that could be construed as a potential conflict of interest.
